# Future Health Today and patients at risk of undiagnosed cancer: a pragmatic cluster randomised trial of quality- improvement activities in general practice

**DOI:** 10.3399/BJGP.2024.0491

**Published:** 2025-04-08

**Authors:** Sophie Chima, Javiera Martinez-Gutierrez, Barbara Hunter, Adrian Laughlin, Patty Chondros, Natalie Lumsden, Douglas Boyle, Craig Nelson, Paul Amores, An Tran-Duy, Jo-Anne Manski-Nankervis, Jon Emery

**Affiliations:** Department of General Practice and Primary Care, University of Melbourne, Melbourne, Australia.; Department of General Practice and Primary Care, University of Melbourne, Melbourne, Australia; Department of Family Medicine, Pontificia Universidad Católica de Chile, Santiago, Chile.; Department of General Practice and Primary Care, University of Melbourne, Melbourne, Australia.; Department of General Practice and Primary Care, University of Melbourne, Melbourne, Australia.; Department of General Practice and Primary Care, University of Melbourne, Melbourne, Australia.; Department of General Practice and Primary Care, University of Melbourne, Melbourne, Australia; Western Health Chronic Disease Alliance, Western Health, Sunshine, Australia.; Department of General Practice and Primary Care, University of Melbourne, Melbourne, Australia; Centre for Research Excellence in Interactive Digital Technology to Transform Australia’s Chronic Disease Outcomes, Melbourne, Australia.; Department of Medicine, Western Health, University of Melbourne, Sunshine, Australia; Department of Nephrology, Western Health, Sunshine, Australia.; Centre for Health Policy, University of Melbourne, Melbourne, Australia; Methods and Implementation Support for Clinical Health Research Hub, University of Melbourne, Melbourne, Australia.; Centre for Health Policy, University of Melbourne, Melbourne, Australia; Methods and Implementation Support for Clinical Health Research Hub, University of Melbourne, Melbourne, Australia.; Department of General Practice and Primary Care, University of Melbourne, Melbourne, Australia; Primary Care and Family Medicine, LKC Medicine, Nanyang Technological University, Singapore.; Department of General Practice and Primary Care, University of Melbourne, Melbourne, Australia, and Western Health, Sunshine, Australia.

**Keywords:** cancer, clinical decision support, diagnosis, general practice, primary care

## Abstract

**Background:**

Diagnosing cancer in general practice is complex, given the non-specific nature of many presenting symptoms and the overlap of potential diagnoses.

**Aim:**

This trial aimed to evaluate the effectiveness of Future Health Today (FHT) — a technology that provides clinical decision support, auditing, and quality-improvement monitoring — on the appropriate follow-up of patients at risk of undiagnosed cancer.

**Design and setting:**

Pragmatic, cluster randomised trial undertaken in general practices in Victoria and Tasmania, Australia.

**Method:**

Practices were randomly assigned to receive recommendations for follow-up investigations for cancer (FHT cancer module) or the active control. Algorithms were applied to the electronic medical record, and used demographic information and abnormal test results that are associated with a risk of undiagnosed cancer (that is, anaemia/iron deficiency, thrombocytosis, and raised prostate-specific antigen) to identify patients requiring further investigation and provide recommendations for care. The intervention consisted of the FHT cancer module, a case-based learning series, and ongoing practice support. Using the intention-to-treat approach, the between-arm difference in the proportion of patients with abnormal test results who were followed up according to guidelines was determined at 12 months.

**Results:**

In total, 7555 patients were identified as at risk of undiagnosed cancer. At 12 months post-randomisation, 76.0% of patients in the intervention arm had received recommended follow-up (21 practices, *n* = 2820/3709), compared with 70.0% in the control arm (19 practices, *n* = 2693/3846; estimated between-arm difference = 2.6% [95% confidence interval (CI)] = −2.8% to 7.9%; odds ratio = 1.15 [95% CI = 0.87 to 1.53]; *P* = 0.332).

**Conclusion:**

The FHT cancer module intervention did not increase the proportion of patients receiving guideline-concordant care. The proportion of patients receiving recommended follow-up was high, suggesting a possible ceiling effect for the intervention.

## Introduction

General practice is the first point of care for most patients diagnosed with cancer.^1^^,^^2^ Diagnosis at an early stage can improve a patient’s chance of successful treatment, reduce healthcare utilisation and associated costs, and improve the patient experience and quality of life.^3^^–^5 GPs manage a cohort of patients who are increasingly complex, and many patients with cancer initially present with limited specific features, which can make diagnosis challenging.^6^ Patients with non-specific symptoms — or symptoms that are suggestive of other, more-common diagnoses — may experience prolonged diagnostic intervals and multiple presentations to the GP.^7^^–^9 The timeliness of diagnosis can be affected by a range of factors, such as a lack of timely access to investigative tests (health-system factors), cognitive or diagnostic errors (physician factors), or delayed follow-up influenced by knowledge or beliefs (patient factors);^10^ these all play a role in the suboptimal follow-up of abnormal test results in primary care, which has been shown to contribute to delays in diagnosing cancer.^11^^,^^12^ Strategies to minimise prolonged diagnostic intervals and reduce missed opportunities for cancer diagnosis in general practice are, therefore, a priority.

Quality-improvement (QI) interventions have the potential to improve the uptake of guideline-based care.^13^ Successful QI programmes are often multifactorial and include complementary components, such as clinical decision support (CDS) and auditing tools.^14^ CDS tools are designed to support GPs in diagnostic decision making — novel technologies can flag patients who may benefit from further follow-up, often at the point of care. Broadly, these have shown promise in helping to both improve the quality of care and adherence to guidelines, and positively affect referral behaviours.^15^^–^17 Auditing systems can complement CDS by applying a safety-netting approach at the practice-population level, thereby facilitating the identification of patients for review, referral, or further investigation based on evidence-based guidelines.^18^^,^^19^ However, the utility of such tools to support the diagnosis of cancer in primary care is unclear: previous studies have been hindered by poor integration with clinical software, disruptions to clinical workflows, and negative GP perceptions.^20^^,^^21^

**Table:** How this fits in

Quality-improvement and clinical decision support (CDS) tools have the potential to help diagnose cancer in general practice. The Future Health Today cancer module provided guideline-based recommendations for patients with abnormal blood-test results that placed them at risk of undiagnosed cancer. Although the intervention did not increase the proportion of patients receiving recommended follow-up, this pragmatic trial highlighted how well routine blood tests are being followed up in primary care. Given the potential ceiling effect of the intervention and the acceptability of the tool in general practice, symptom-based CDS for cancer diagnosis should be explored.

In this trial, the cancer module of Future Health Today (FHT), a QI and CDS tool, was implemented with recommendations for guideline-based follow-up in patients with abnormal blood-test results that placed them at risk of undiagnosed cancer. The aim was to determine whether the FHT cancer module, coupled with education, training, and practice support, increased the proportion of patients receiving guideline-based care at 12 months post-randomisation, compared with an active control.

## Method

### Design

A pragmatic, stratified, cluster randomised trial was conducted in Australian general practice from October 2021 until September 2022. Practices were randomly allocated to participate in either the intervention (FHT cancer module) arm or the active control (pharmacological management in people with chronic kidney disease [CKD] — the CKD module) arm. The study protocol has been published online and on the Australia and New Zealand Clinical Trial Registry (ANZCTR) (reference: ACTRN12620000993998). Results for the active control intervention will be reported separately.

The CKD module was chosen for the active control arm as it was one of the first FHT modules developed and underwent a rigorous co-design process. The module targeted a different disease and patient population, with no overlap in recommended interventions, to ensure minimal contamination (the CKD module targeted patients aged 18–80 years, with a recorded diagnosis of, or pathology consistent with, CKD that might benefit from pharmacological therapy to reduce risk of developing cardiovascular disease).^22^^,^^23^ An active control arm was used to account for the intervention effect of delivering a QI programme in general practice.

### Study population

Practices were recruited in two Australian states, Victoria and Tasmania, via national newsletters and two practice-based research networks, and interested practices were assessed for eligibility. The inclusion and exclusion criteria are given in Supplementary Box S1.

Practices were required to have at least one computer with a Windows 10 operating system so the data-extraction software (GRHANITE) could be installed,^24^ and had to supply written consent to participate. They were compensated AU$2250 for participation in the trial and reimbursed at two time points: after installation of the technology and at the end of the trial. Reimbursement was not used as an incentive for general practice staff to use the tool.

As the outcomes were determined using de-identified electronic medical record (EMR) data from practices, no individual patient-level consent was required. Inclusion criteria for patients are described in Table 1. The authors included all patients identified as having one of the abnormal blood-test results associated with a risk of undiagnosed cancer at baseline and during the first 6 months of the trial (referred to as the closed and open cohorts, respectively).

**Table 1.** Recommendation criteria and recommended follow-up by abnormal test result type

**Test**	**Recommendation criteria**	**Exclusion criteria**	**Guideline-based follow-up^a^**	**Cancer type**
Anaemia/iron deficiency	Men and women aged 50–80 years Haemoglobin <130 g/L in men and <115 g/L in women or MCV <80 fl or MCH <27 pg or ferritin <30 µg/L in previous 6 months	Recorded colorectal, gastrointestinal, endometrial, unspecified, or metastatic cancer in last 5 years	Repeat FBC or iron studies Coeliac disease serology FOBT Colonoscopy Transvaginal ultrasound Review of relevant symptoms^b^ Prescription of an oral supplement or iron infusion Referral to gastroenterologist	Colorectal Gastrointestinal Endometrial
Thrombocytosis	Males and females aged 40–80 years Raised platelet count (>400 × 10^9^/L) in previous 6 months	Recorded diagnosis of lung, colorectal, gastro-oesophageal, ovarian, endometrial, or unspecific cancer in previous 5 years	Repeat platelet count Chest X-ray Chest CT FOBT CA-125 Transvaginal ultrasound Review of relevant symptoms^b^ Referral to gastroenterologist	Colorectal Gastrointestinal Lung Endometrial Ovarian
Raised PSA	Men aged 40–80 years PSA: >2.0 ng/ml (40–49 years); >3.0 ng/ml (≥50 years) in previous 6 months	Recorded diagnosis of prostate cancer	Repeat PSA and/or free-to-total PSA percentage Referral to urologist	• Prostate

a *The recommendations and suggested investigations varied depending on age, sex, and number of abnormal results.*

b *Complete list of symptoms outlined in Supplementary Box S2. The authors did not include symptoms of prostate cancer as it is not considered a first-line investigation for patients with raised PSA. CA-125 = cancer antigen 125. CT = computed tomography. FBC = full blood count. FOBT = faecal occult blood test. MCH = mean corpuscular haemoglobin. MCV = mean cell volume. PSA = prostate-specific antigen.*

### Intervention

The intervention comprised the FHT software, educational sessions, benchmarking reports, and practice support. The software consists of a CDS (which provides recommendations at the point of care) and a web-based portal (which contains an audit tool, QI monitoring, and educational resources). It was co-designed and tested with general practice clinical staff and consumers before the trial, as outlined by Hunter *et al*.^25^ The FHT software is integrated with the practice EMR and identifies patients who may benefit from guideline-informed care. Disease-specific modules are developed for use in FHT. The FHT cancer module used patient information (age, sex, previous cancer diagnosis) and recent blood-test results (iron-deficiency/anaemia, raised prostate-specific antigen (PSA), thrombocytosis [Table 1]). If a patient was identified as requiring further follow-up, the clinician received recommendations at the point of care and in the audit tool, underpinned by national and international guidelines.^26^^–^30 Algorithms embedded within the FHT software ran each night, updating patient-specific recommendations and identifying new patients at risk. Examples of the CDS and audit tool can be found in Supplementary Figures S1a and S1b.

In addition to the FHT cancer module, the intervention included:

- an option to participate in six project ECHO educational sessions — namely, 1-hour webinars that included a didactic presentation, case discussion, and an open discussion;^31^
- quarterly benchmarking reports to enable a review of practices’ progress and to compare their progress with other practices receiving the same intervention; and
- a study coordinator who provided training and ongoing support throughout the trial.

This composite implementation strategy was informed by the Reach, Effectiveness, Adoption, Implementation, and Maintenance (RE-AIM) framework.^32^

The FHT software was installed on at least one computer in each practice prior to trial commencement. Practices were asked to generate a list of patients who were flagged by the cancer module on the first day of the trial and again at the 6-month mark, using the audit tool, so that benchmarking information could be determined. After generating these lists, practices were invited to use FHT as they chose during the trial.

### Randomisation and blinding

Randomisation was carried out by a trial statistician who was not involved in the trial recruitment or data collection. After all practices were recruited and baseline measures collected, a computer-generated schedule randomised practices on a 1:1 ratio to each arm. Randomisation was stratified by Index of Relative Socioeconomic Disadvantage (IRSD) terciles^33^ and the number of full-time equivalent (FTE) GPs (≤4 versus >4), using random permuted block sizes of two and four within stratum.

It was not possible to blind general practice staff who received the intervention or the research team providing support. Practices were blinded to the clinical topic of the active control arm to ensure that the risk of contamination was limited. The statistician overseeing the analysis and investigators not involved in practice support remained blinded until after the data were analysed and the results were interpreted.

### Outcomes

The primary outcome was the proportion of patients identified as being at risk of undiagnosed cancer based on abnormal blood-test results, who were assessed or investigated according to national and international guidelines.^26^^–^30 If a patient had more than one altered test result, guideline-based follow-up for either abnormal test was considered appropriate follow-up. If there was no record of an appropriate investigation, symptom recorded, or relevant referral in the EMR, patients were considered to have not received guideline-based follow-up.

Secondary outcomes included the:

- proportion of patients who received guideline-based follow-up dependent on the type of altered test result;
- proportion of patients treated for iron deficiency;
- proportion of patients with a relevant diagnosis of cancer;
- rate of general practice encounters;
- time to first investigation; and
- cost incurred by the patient.

Outcomes were measured at 12 months post-randomisation using EMR data. No patient-level data were collected through FHT. Data were extracted from the practice management software database on a nightly basis and processed locally by applying FHT algorithms; as such, a separate data-extraction platform — GRHANITE — was used. GRHANITE allowed for the extraction of de-identified data from EMRs, after which it was curated and stored in Patron, a general practice data repository,^34^ allowing for assessment of the trial outcomes. Algorithms that were identical to those executed by the FHT platform were run within the Patron dataset. This replication allowed for identification of the same eligible patients to determine baseline measures and patient outcomes for analysis.

The low incidence of cancer in general practice precluded new cancer diagnoses as the primary outcome. The trial was powered to detect a behavioural outcome reflecting the mechanism to improve cancer diagnoses in general practice.

### Sample size

Assuming that 30% of patients were managed appropriately in the active control arm, an estimated 1200 eligible patients (an average of 30 per practice) were needed to detect an absolute 20% increase in the percentage of patients at risk of an undiagnosed cancer who received guideline-based follow-up in the intervention arm.^35^^,^^36^ Sample sizes were based on achieving 99% power, for a two-sided 5% significance level, an intra-cluster correlation of 0.03 to account for the effect of clustering at the practice level, and a coefficient of variation of 0.41 to allow for variable cluster sizes. The intra-cluster correlation and coefficient of variation were estimated using the condition-specific patient cohorts in 77 general practices available in the Patron dataset.^34^ The authors allowed for the attrition of four practices by 12 months. The sample size calculation was conducted using Stata (version 17.0).

### Statistical methods

For the primary outcome and binary secondary outcomes, the odds ratio (OR) and the difference in proportions were estimated using a generalised linear regression model, with logit and identity link functions, respectively. To allow for the correlation of outcomes in general practice, generalised estimating equations were used with an exchangeable correlation structure and robust standard errors (SEs). An intention-to-treat approach was used; all patients flagged for follow-up were included in each analysis regardless of intercurrent events (death, pregnancy). Randomisation stratification factors (namely, IRSD terciles and number of FTE GPs) were included as variables in all analyses.

The between-arm difference in the rate of consultations per year and the rate ratio of visits were modelled using a negative binomial mixed-effects model, with fixed effects for the study arm and randomisation stratification factors, and random effects for general practice. A Cox proportional hazards analysis was conducted to examine the between-arm differences in the time to first investigation in the open cohort, adjusting for randomisation stratification factors and using robust SEs. Planned economic analyses are described in Supplementary Text S1. Sensitivity analyses included:

- adjusting for confounders measured at baseline (patient age, sex, practice participation in a formalised QI programme, and whether patients were identified in the open or closed cohort) (all outcomes);
- adjusting the outcome definition to exclude symptoms (primary outcome only); and
- using other prostate diagnoses as a proxy for PSA referrals (secondary outcome relating to PSA follow-up only).

Analyses were conducted using Stata (version 17.0). A statistical analysis plan is available on ANZCTR (reference: ACTRN12620000993998).

## Results

**Figure 1.**
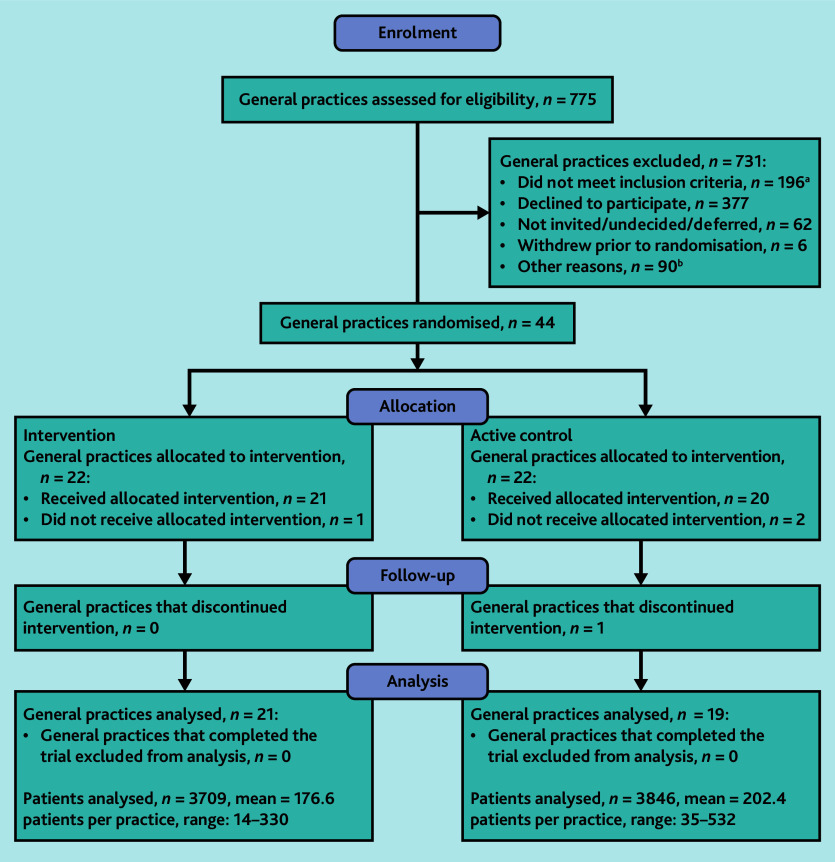
CONSORT flow diagram. ^a^Frequent inclusion criteria that were not met included practice size, willingness to install GRHANITE, and incompatible practice software. ^b^Other reasons for exclusion included recruitment targets being met in the trial and practices being lost to follow-up/non-responsive.

Practice recruitment occurred between October 2020 and August 2021. Eligibility was assessed in 775 practices, from which 50 practices were recruited. Six practices withdrew prior to randomisation, leaving 44 practices randomised (Figure 1). Three practices withdrew after randomisation but before trial initiation, and one practice withdrew during the trial. Recruitment in Tasmania was commenced in response to challenges related to COVID-19 in Victoria. Only one practice in Tasmania consented before the recruitment target was met.

In total, 7555 patients were identified as being at risk of undiagnosed cancer; 3709 of these were in the intervention arm and 3846 were in the active control arm. Baseline characteristics of the 40 practices and their staff demographics are presented in Table 2; characteristics of the eligible patients are presented in Table 3.

**Table 2.** Baseline characteristics of practices and practice staff

**Characteristics**	**Total**	**Intervention**	**Control**
**Practices**			
Total, *n*	40	21	19
State, *n* (%)			
Victoria	39 (97.5)	20 (95.2)	19 (100.0)
Tasmania	1 (2.5)	1 (4.8)	0 (0.0)
Index of Relative Socioeconomic Disadvantage tercile, *n* (%)			
1 — most disadvantaged	12 (30.0)	6 (28.6)	6 (31.6)
2	12 (30.0)	6 (28.6)	6 (31.6)
3 — least disadvantaged	16 (40.0)	9 (42.9)	7 (36.8)
Previously participated in QI programme, *n* (%)	18 (45.0)	9 (42.9)	9 (47.4)
Practice size, *n* (%)			
≤4 FTE GPs	21 (52.5)	12 (57.1)	9 (47.4)
>4 FTE GPs	19 (47.5)	9 (42.9)	10 (52.6)
Eligible patients per practice, mean (range)	188.9 (14–532)	176.6 (14–330)	202.4 (35–532)
FTE GPs^a^	4.5 (3.5–6.0)	4.0 (3.5–5.5)	4.5 (3.5–7.5)
FTE nurses^a^	1.8 (1.0–2.5)	2.0 (1.5–2.5)	1.5 (1.0–3.0)
FTE practice managers/administrative staff^a^	4.0 (3.0–5.0)	3.5 (2.5–4.5)	4.5 (3.5–6.0)

**GPs**			
Total, *n*	309	145	164
Gender, *n* (%)			
Male	178 (57.6)	85 (58.6)	93 (56.7)
Female	131 (42.4)	60 (41.4)	71 (43.3)
Age, *n* (%)^a^			
<35 years	40 (15.0)	21 (15.8)	19 (14.2)
35–50 years	129 (48.3)	73 (54.9)	56 (41.8)
>50 years	98 (36.7)	39 (29.3)	59 (44.0)

**Practice nurses**	127	63	64
Gender, *n* (%)			
Male	7 (5.5)	4 (6.3)	3 (4.7)
Female	120 (94.5)	59 (93.7)	61 (95.3)
Age, *n* (%)			
<35 years	65 (51.2)	32 (50.8)	33 (51.6)
35–50 years	31 (24.4)	17 (27.0)	14 (21.9%)
>50 years	31 (24.4)	14 (22.2)	17 (26.6%)

**Practice managers/administrative staff**	257	127	130
Gender, *n* (%)			
Male	27 (10.5)	13 (10.2)	14 (10.8)
Female	230 (89.5)	114 (89.8)	116 (89.2)
Age, *n* (%)^a^			
<35 years	130 (51.6)	55 (43.3)	75 (60.0)
35–50 years	59 (23.4)	33 (26.0)	26 (20.8)
>50 years	63 (25.0)	39 (30.7)	24 (19.2)

a *Median and IQR.*

b *Percentages based on number of staff who provided age data: GPs,* n *= 267 (87%); practice managers,* n *= 252 (98%). FTE = full-time equivalent. IQR = interquartile range.*

**Table 3.** Baseline characteristics of patients

**Characteristic**	**All patients**		**Closed cohort^a^**		**Open cohort^b^**	

	**Intervention**	**Control**	**Intervention**	**Control**	**Intervention**	**Control**
Total, *n*	3709	3846	2141	2280	1568	1566
Sex, *n* (%)						
Male	1840 (49.6)	1946 (50.6)	1067 (49.8)	1173 (51.4)	773 (49.3)	773 (49.4)
Female	1869 (50.4)	1900 (49.4)	1074 (50.2)	1107 (48.6)	795 (50.7)	793 (50.6)
Age in years, mean (SD)	64.7 (10.5)	64.5 (10.3)	65.2 (10.4)	65.1 (10.3)	64.0 (10.5)	63.6 (10.2)

Marker of anaemia, *n* (%)	2553 (68.8)	2555 (66.4)	1494 (69.8)	1530 (67.1)	1059 (67.5)	1025 (65.5)
Raised platelets, *n* (%)	767 (20.7)	826 (21.5)	436 (20.4)	499 (21.9)	331 (21.1)	327 (20.9)
Altered PSA, *n* (%)	666 (18.0)	773 (20.1)	381 (17.8)	466 (20.4)	285 (18.2)	307 (19.6)

Two altered tests, *n* (%)						
Iron-deficiency and thrombocytosis	176 (4.7)	221 (5.7)	104 (4.9)	152 (6.7)	72 (4.6)	69 (4.4)
Iron deficiency and raised PSA	93 (2.5)	72 (1.9)	61 (2.8)	55 (2.4)	32 (2.0)	17 (1.1)
Raised PSA and thrombocytosis	14 (0.4)	23 (0.6)	9 (0.4)	14 (0.6)	5 (0.3)	9 (0.6)
Three altered tests, *n* (%)	6 (0.2)	8 (0.2)	4 (0.2)	6 (0.3)	2 (0.1)	2 (0.1)

a *Patients identified as at risk of undiagnosed cancer at trial initiation.*

b *Patients identified as at risk of undiagnosed cancer during the first 6 months of the trial. PSA = prostate-specific antigen. SD = standard deviation.*

### Primary outcome

**Figure 2.**
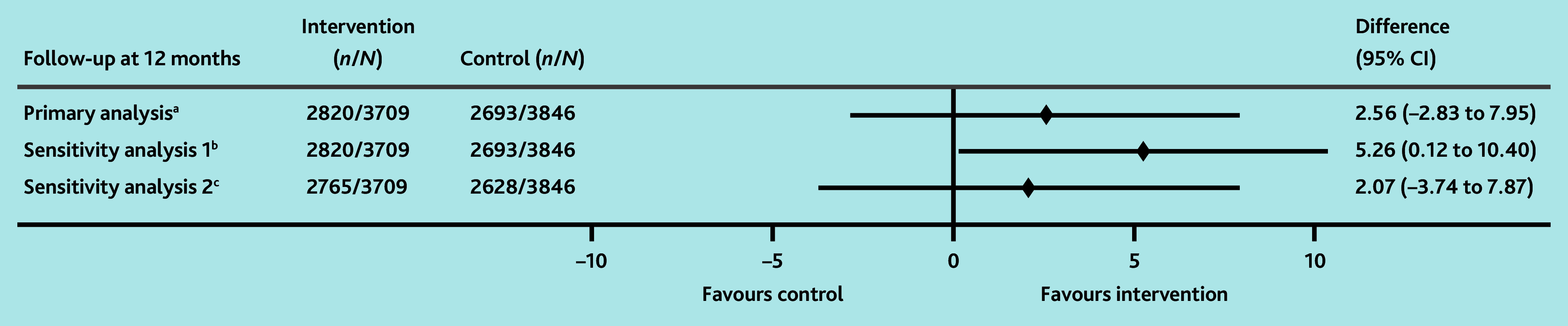
Forest plot showing estimated intervention effects (absolute differences), including planned sensitivity analyses for the primary outcome. ^a^ Difference in the proportion of patients followed up according to guidelines at 12 months between the intervention and the control arms. ^b^Sensitivity analysis adjusted for the practice participation in formalised quality improvement programme, patient’s age in years, cohort type, and patient’s sex. ^c^Sensitivity analysis without symptoms. *N* = number of investigations and *n* = number or participants.

At 12 months, 76.0% of patients in the intervention arm were followed up according to guidelines (21 practices, 2820 patients), compared with 70.0% in the control (19 practices, 2693 patients), with an estimated difference of 2.6% (95% CI = −2.8% to 7.9%) (Table 4). A planned sensitivity analysis to adjust for confounders identified at baseline showed an absolute difference of 5.3% (95% CI = 0.1% to 10.4%) in favour of the intervention (Figure 2), but the 95% CIs of the OR for this difference crossed unity (OR = 1.12, 95% CI = 0.87 to 1.45) (Table 4). To account for the non-specific nature of some symptoms associated with cancer, the authors conducted a sensitivity analysis excluding the assessment of symptoms as a measure of appropriate follow-up. This demonstrated similar results to the primary analysis (difference = 2.1%, 95% CI = −3.7% to 7.9%) (Table 4).

**Table 4.** Primary and secondary outcomes

	**All participants**	**Intervention**	**Control**	**Estimated intervention effect**		***P*-value**
**Primary outcome**						

**Guideline-based follow-up**				**Diff (95% CI)**	**OR (95% CI)**	
Participants, *n*	7555	3709	3846			
Primary analysis, *n* (%)	5507 (72.9)	2820 (76.0)	2693 (70.0)	2.6 (−2.8 to 7.9)	1.15 (0.87 to 1.53)	0.332
Sensitivity analysis^a^				5.3 (0.1 to 10.4)	1.12 (0.87 to 1.45)	0.388
Sensitivity analysis^b^	5393 (71.4)	2765 (74.5)	2628 (68.3)	2.1 (−3.7 to 7.9)	1.12 (0.83 to 1.50)	0.465

**Secondary outcomes^c^**						

**Follow-up of iron deficiency**				**Diff (95% CI)**	**OR (95% CI)**	
Partipants, *n*	5108	2553	2555			
Primary analysis, *n* (%)	3921 (76.8)	2031 (79.6)	1890 (74.0)	2.7 (−3.4 to 8.8)	1.18 (0.84 to 1.68)	0.342
Sensitivity analysis^a^				5.2 (−0.3 to 10.7)	1.10 (0.80 to 1.53)	0.556

**Prescriptions for iron deficiency**				**Diff (95% CI)**	**OR (95% CI)**	
Partipants, *n*	5108	2553	2555			
Primary analysis, *n* (%)	575 (11.3)	327 (12.8)	248 (9.7)	2.9 (−0.03 to 5.8)	1.31 (0.97 to 1.76)	0.077
Sensitivity analysis^a^				2.5 (−0.04 to 5.0)	1.25 (0.92 to 1.70)	0.160

**Follow-up of raised platelets**				**Diff (95% CI)**	**OR (95% CI)**	
Partipants, *n*	1593	767	826			
Primary analysis, *n* (%)	1066 (66.9)	535 (69.8)	531 (64.3)	3.8 (−3.2 to 10.7)	1.20 (0.87 to 1.66)	0.266
Sensitivity analysis^a^				6.9 (0.7 to 13.1)	1.33 (0.95 to 1.87)	0.100

**Follow-up of raised PSA**				**Diff (95% CI)**	**OR (95% CI)**	
Partipants, *n*	1439	666	773			
Primary analysis, *n* (%)	923 (64.1)	457 (68.6)	466 (60.3)	1.9 (−7.6 to 11.5)	1.08 (0.71 to 1.66)	0.723
Sensitivity analysis^a^				4.3 (−4.7 to 13.2)	1.23 (0.82 to 1.83)	0.315
Sensitivity analysis, *n* (%)^d^	940 (65.3)	463 (69.5)	477 (61.7)	1.2 (−8.2 to 10.7)	1.05 (0.69 to 1.60)	0.823

**Cancer diagnosis**				**Diff (95% CI)**	**OR (95% CI)**	
Partipants, *n*	7555	3709	3846			
Primary analysis, *n* (%)	56 (0.7)	26 (0.7)	30 (0.8)	0.1 (−2.0 to 2.2)	0.86 (0.41 to 1.79)	0.686

**Rates of encounters**				**Diff (95% CI)**	**RR (95% CI)**	
Partipants, *n*	7555	3709	3846			
Visits, *n*	80 869	43 592	37 277			
Primary analysis, mean (SD)	10.7 (11.4)	11.8 (12.6)	9.7 (10.0)	0.15 (−0.07 to 0.37)	1.16 (0.93 to 1.45)	0.192
Sensitivity analysis^a^				0.15 (−0.07 to 0.38)	1.17 (0.93 to 1.46)	0.173

**Time to follow-up**					**HR (95% CI)**	
Partipants, *N*/*n*	2189/3134	1134/1568	1055/1566			
Primaryanalysis, median (95% CI)^e^	91.0 (79.0 to 104.0)	81.0 (62.0 to 100.0)	100.0 (83.0 to 120.1)		1.14 (0.98 to 1.32)	0.090
Sensitivity analysis^f^					1.14 (0.98 to 1.32)	0.090

a *Sensitivity analysis additionally adjusted for the practice participation in formalised QI programme, patient’s age in years, cohort type, and patient’s sex.*

b *Sensitivity analysis, as described for primary analysis, without symptoms.*

c *Primary analysis for binary outcomes: generalised linear regression using generalised estimating equations with robust standard errors, adjusted for GP, full-time equivalent, and Index of Relative Socioeconomic Disadvantage tercile.*

d *Sensitivity analysis, as described for primary analysis, with a proxy for PSA referrals.*

e *Median time to investigation estimated using the Kaplan–Meier estimator. For follow-up events that occurred on the same day that abnormal tests were received, a small time variation was applied (0.01 days), so that all patients flagged for follow-up were included in the time to follow-up analysis.*

f *Sensitivity analysis adjusted for the practice participation in formalised QI programme, patient’s age in years, and patient’s sex. Diff = difference in proportions between the two arms. HR = hazard ratio. OR = odds ratio. PSA = prostate-specific antigen. QI = quality improvement. RR = rate ratio. SD = standard deviation. SE = standard error.*

### Secondary outcomes

Results of the secondary outcomes are detailed in Table 4 and Supplementary Text S1. There was insufficient evidence of an effect of the intervention by type of abnormal test result. For patients with thrombocytosis, the effect seen in the planned sensitivity analysis (after adjusting for confounders) was similar to that of the primary analysis, with a small absolute difference (difference = 6.9%, 95% CI = 0.7% to 13.1%) (Table 4).

For patients who were flagged with a raised PSA, a sensitivity analysis using other prostate diagnoses as a proxy for receiving a referral to a specialist was conducted, but the results remained unchanged (difference = 1.2%; 95% CI = −8.2% to 10.7%) (Table 4).

There was no statistical evidence of an effect of the intervention compared with the active control on the proportion of patients diagnosed with cancer, the rates of general practice encounters, time to first investigation, or healthcare costs (Supplementary Text S1).

## Discussion

### Summary

In this pragmatic trial, the use of a novel QI and CDS tool did not increase the proportion of patients with abnormal test results that might have been indicative of an undiagnosed cancer receiving recommended follow-up. Although the planned sensitivity analyses adjusting for confounders showed some limited evidence of differences in the primary outcome and the follow-up of raised platelets, these differences were small and may not be clinically important. The proportion of patients receiving recommended follow-up was high in all practices; >70% of patients flagged by the tool had recorded follow-up investigations during the study period. Although this could indicate a potential ceiling effect that may have affected the findings, the results nevertheless demonstrate the high level of guideline-concordant care for such patients in Australian general practice.

### Strengths and limitations

This large, highly powered study demonstrates the use of a QI and CDS tool in a real-world setting. As a pragmatic study, GPs were able to use the tool as and when they wanted, which reflects the way it would be used in everyday practice.

The trial was conducted during the COVID-19 pandemic (in which there were >260 days of lockdown in Victoria, with restrictions placed on how, and when, people could leave their homes) and continued through a nationwide COVID-19 immunisation roll-out in general practice. The burden on general practice was high and there was a shift in usual care, with many consultations conducted via telehealth.^37^ The shift in priorities, the time and resource cost, and the associated rates of burnout would have contributed to each practice’s capacity to participate in the intervention;^38^^,^^39^ nevertheless, the performance of practices involved in the trial was much higher than reported in international literature.^35^^,^^36^

A potential ceiling effect has been described, due to the proportion of patients followed up across both arms being high. It is plausible that the practices that agreed to participate in a QI-based intervention were high-performing practices because they were already interested, skilled, or conducting QI in their practice. This may, therefore, limit the generalisability of the findings to the wider general practice population. The mobility of practice staff — in particular, practice managers — may have led to the unblinding of the clinical topic of the active control arm; however, the authors believe the occurrence of unblinding was uncommon, with only one confirmed report of a practice manager working across multiple practices involved in the trial.

EMR data were used to determine the study outcomes, which the authors consider to be both a strength and a limitation. A rigorous method in defining and identifying follow-up was used, creating a comprehensive list of terms used in the EMR relating to symptoms, referrals, and tests that made up the primary outcome. However, EMR data are not collected primarily for research purposes, and this has implications for the quality and completeness of the data.^40^^,^^41^ The authors were only able to capture information from extractable fields (for example, reason for encounter) and it is likely they have missed some symptoms that were recorded as free text in the clinical notes and referrals, and not labelled with the referring specialty. This is especially important for the follow-up of PSA: the definition of guideline-based follow-up included only a repeat PSA test or referral to urologist, and it is likely that the proportion of follow-up across both arms was underestimated because of limited referral data.

### Comparison with existing literature

The proportion of patients followed up according to guidelines was higher than reported in international studies:^35^^,^^36^ one found that <50% of patients with iron deficiency were adequately followed up, whereas the other demonstrated that 40% of patients had no follow-up tests at all.^35^ In total, >75% of patients in the study presented here who were identified as having iron deficiency during the study period received guideline-based follow-up. The proportion of guideline-based follow-up was also high for raised platelets, despite the relatively new evidence from primary care on the association between raised platelets and cancer risk.^42^ It is important to acknowledge, however, that this higher proportion of follow-up may, in part, be due to the easy access to routine blood tests in Australian general practice.

The results of this trial were in contrast to those of a similar US-based study, in which an electronic trigger was used to identify patients with abnormal findings (relating to faecal occult blood test [FOBT], PSA, iron-deficiency anaemia, and haematochezia), resulting in a significant reduction in the time to diagnosis for prostate and colorectal cancer.^12^ These differences may be due to health-system differences (for example, access to routine blood tests and follow-up investigations) and the tests considered (for example, an FOBT, which would indicate a likelihood that GPs would be actively looking for, or considering, cancer).

A process evaluation was embedded in the trial reported here to explore the successes and failures of implementation. These results will be reported separately (paper in submission), but the key findings give context to the study results. Two key implementation barriers were identified:

- uptake of the supportive components of the intervention was low, aside from some initial training on the software (for example, attendance at the ECHO sessions ranged from three to nine participants); and
- practice characteristics and contextual factors meant there was limited ability for some practices to engage with the tool when their patient population was not suited to the FHT module that was implemented (for example, younger or transient patient demographics of some inner-city practices).

Low uptake and adoption of CDS tools are rarely down to a single implementation issue — instead, these may be driven by multiple, interrelated, dynamic factors, including organisational, cultural, and technological challenges. Previous studies have highlighted how design and implementation failures can affect patient care: prompt fatigue, task complexity, and increased cognitive load are just a few of the mechanisms by which CDS can disrupt the workflow.^43^^,^^44^ The challenges that were identified here, with regard to implementing CDS and audit tools, along with the complexities in reaching the entire practice, are consistent with those found in previous studies,^43^^,^^45^ in which it has been shown that similar interventions have resulted in low use and uptake^20^^,^^45^ or have failed to show an effect.^36^^,^^46^

### Implications for research and practice

Although the results did not indicate a large intervention effect, interviews with some of the practice staff concluded that the tool was considered useful and acceptable to GPs (results published separately).^47^ Given the potential ceiling effect in this study, future FHT modules or future CDS tools developed to support the diagnosis of cancer may be better suited to investigations or symptom presentations with lower rates of follow-up. It is known that the management of non-specific symptoms in primary care is a contributor to delays in diagnosis^48^ and it may, therefore, be useful for future CDS to include symptom-based recommendations that are able to capture new and ongoing symptoms, and provide patient-specific recommendations. Similarly, given the acceptability of the tool, the effectiveness of other FHT modules — that is, those for different diseases and/or investigations — should be explored. For the FHT cancer module, future work could evaluate the effectiveness of the module in practices with specific patient populations or in a subset of lower-performing practices.

## Data

The data that support the findings of this study are available from the PATRON Primary Care Research Data Repository, subject to successful application process.
